# Liquid‐Phase Hot Deformation to Enhance Thermoelectric Performance of n‐type Bismuth‐Telluride‐Based Solid Solutions

**DOI:** 10.1002/advs.201901702

**Published:** 2019-09-14

**Authors:** Yehao Wu, Yuan Yu, Qi Zhang, Tiejun Zhu, Renshuang Zhai, Xinbing Zhao

**Affiliations:** ^1^ State Key Laboratory of Silicon Materials and School of Materials Science and Engineering Zhejiang University Hangzhou 310027 China; ^2^ I. Physikalisches Institut (IA) RWTH Aachen University 52074 Aachen Germany

**Keywords:** bismuth tellurides, hot deformation, thermoelectric materials, thermoelectric properties

## Abstract

Bismuth‐telluride‐based solid solutions are the best commercial thermoelectric materials near room temperature. For their n‐type polycrystalline compounds, the maximum figures of merit (*zT*s) are often less than 1.0 due to the degraded carrier mobility resulting from the loss of texture. Herein, a liquid‐phase hot deformation procedure, during which the Bi_2_(Te,Se)_3_ ingots are directly hot deformed with the extrusion of liquid eutectic phase, is performed to enhance the thermoelectric performance of n‐type Bi_2_(Te,Se)_3_ alloys. The deformation‐induced dynamic recrystallization is remarkably suppressed due to the reduction of nucleation sites and the release of deformation stress by liquid phase, contributing to a weakened carrier scattering and enhanced carrier mobility. The liquid eutectic phase also facilitates the rotation of grains and enhanced (000*l*) texture, further improving carrier mobility. In addition, the dense dislocations and lattice distortion introduced into the matrix reduce the lattice thermal conductivity. As a result, a high *zT* value of 1.1 at 400 K is obtained, about 75% increment over the normal one‐step hot deformed alloys. This work not only demonstrates a simple and efficient technique for achieving superior n‐type Bi_2_Te_3_‐based materials, but also elucidates the important role of liquid eutectic phase in hot deformation.

## Graphical abstract

Here, liquid‐phase hot deformation is introduced to n‐type polycrystalline Bi_2_Te_3_‐based alloys. Benefitting from the suppression of recrystallization, improvement of texture, and enhancement of phonon scattering, contributing to boosted carrier mobility and reduced lattice thermal conductivity, a peak *zT* ≈ 1.1 at 400 K is successfully obtained for Bi_2_(Te,Se)_3_.

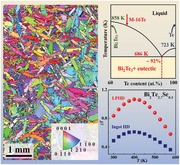

## 1 Introduction

Thermoelectric (TE) materials, which enable direct conversion between heat and electrical energy, have drawn much attention in the past decades. Their conversion efficiency is governed by the dimensionless figure of merit *zT* = α^2^ *σT*/(κ_e_ + κ_L_), where α, σ, *T*, κ_e_, and κ_L_ are the Seebeck coefficient, electrical conductivity, absolute temperature, electronic thermal conductivity, and lattice thermal conductivity, respectively.^1^ Several effective strategies such as carrier concentration optimization,[qv: 1b,2] band engineering,^3^ and phonon engineering^4^ have been widely utilized to boost the material's electrical properties or to reduce κ_L_, resulting in a remarkable improvement of *zT*.^5^

As the state‐of‐the‐art TE materials, bismuth‐telluride‐based solid solutions have been the best commercial materials for solid‐state cooling near room temperature. Both p‐ and n‐type zone‐melted (ZM) Bi_2_Te_3_‐based alloys exhibit *zT* ≈ 1 around room temperature.^5^ Nevertheless, their easy‐cleavage behavior increases the cost of device fabrication. In the past decade, powder metallurgical processes, in which the samples are prepared by mechanical alloying, ball milling (BM), melt spinning, or solvothermal synthesis followed by hot pressing (HP) or spark plasma sintering (SPS), have been widely used to improve the TE performance as well as the mechanical properties for Bi_2_Te_3_‐based alloys. For p‐type (Bi,Sb)_2_Te_3_ materials, a peak *zT* > 1.2 can be readily realized by nanostructuring.[qv: 4d,i,6] Recently, the liquid‐phase sintering (LPS) process, which involves sintering under conditions where solid grains are surrounded by the wetting liquid,^7^ is also successfully applied to enhance the TE properties of p‐type Bi_2_Te_3_‐based alloys.[qv: 2a,4e,8] Nanoscale defects such as dense dislocations[qv: 4e] or Sb‐rich region[qv: 8b] could be introduced during LPS and play important roles in reducing κ_L_. In addition, the texture could be enhanced after LPS,[qv: 8a] which is beneficial for the improvement of carrier mobility *µ*_H_.

For n‐type Bi_2_(Te,Se)_3_ alloys, the LPS process has also been employed to reduce κ_L_ via introducing nanostructures into the matrix. Through LPS, a peak *zT* ≈ 0.98 at 370 K and a peak *zT* ≈ 0.7 at 398 K have been obtained by Zhang et al. and Ge et al., respectively.[qv: 8b,9] However, compared to the p‐type counterparts, *zT*s in n‐type polycrystalline Bi_2_(Te,Se)_3_ alloys are usually less than 1.0 due to the deteriorated texture and the deviation from the optimal carrier concentration resulting from the pulverization of ingots and donor‐like effect.^10^ Recently, Park et al. prepared phase‐pure n‐type K_0.06_Bi_2_Te_3.18_ alloys via the hydrothermal method combined with SPS and a high *zT* > 1.1 at 323 K was achieved.^11^ Hong et al. performed a microwave‐assisted solvothermal method to get high‐quality Bi_2_Te_3−_ *_x_*Se*_x_* nanoplates and obtained a *zT* of 1.23 at 480 K for the sintered sample.^12^ However, these two methods are too complicated to be competitive for a large‐scale production.

In recent years, the hot deformation (HD) process has been successfully applied to enhance texture as well as reduce κ_L_ in n‐type Bi_2_Te_3_‐based materials. By applying a twice HD on n‐type ZM Bi_2_Te_2.79_Se_0.21_ ingot, Hu et al. induced multiscale microstructures into the matrix to strongly scatter phonons and obtained a peak *zT* ≈ 1.2 at 357 K.^13^ Similarly, Li et al. reduced room temperature κ_L_ from 1.2 to 0.8 W m^−1^ K^−1^ and reported on a peak *zT* ≈ 1.1 at 625 K in the hot deformed Bi_1.85_In_0.15_Te_2_Se alloys.^14^ Resulting from the increased *µ*_H_ by texturing in the HD process, peak *zTs* varying from 1.04 to 1.3 can be realized in different n‐type HD Bi_2_Te_3_‐based materials.[qv: 2b,15] Note that, most of them with peak *zT* ≥ 1.1 were obtained by multiple HD. Nevertheless, such an HD procedure is complicated, which includes ingot pulverization by BM, powder sintering by HP or SPS, and repeated hot deformation (up to three times), leads to considerable energy consumption. Meanwhile, the uncontrollable volatilization of Te or Se during multiple high‐temperature processing and the severely increased *n*_H_ caused by the donor‐like effects during ingot pulverization[qv: 2b,10f,15b] could also result in poor repeatability in the HD Bi_2_Te_3_‐based alloys. Hence, it is necessary to develop a new technique with a simpler and milder process to prepare superior n‐type Bi_2_(Te,Se)_3_ materials.

Herein, we report a novel liquid‐phase hot deformation (LPHD) technique to enhance the TE properties of n‐type Bi_2_(Te,Se)_3_ alloys. The lamellate Te‐rich eutectic phase is introduced into the Bi_2_(Te,Se)_3_‐melted ingot. This ingot is then directly hot‐deformed in a larger graphite die at a temperature above the eutectic point. In this case, the Bi_2_(Te,Se)_3_ solid grains are initially surrounded by the liquid eutectic phase and then gradually deformed with the extrusion of liquid. Compared to the multiple HD procedure, the process flow in LPHD technique is much shortened. During one‐step LPHD, the deformation‐induced dynamic recrystallization is remarkably suppressed due to the reduction of nucleation sites at interfaces and the release of deformation stress in the matrix, contributing to a weakened electron scattering and hence an enhanced carrier mobility *µ*_H_. In addition, the *µ*_H_ is further improved by texturing during LPHD. Meanwhile, a significant reduction of the lattice thermal conductivity is also realized in the LPHD sample as a result of the enhanced phonon scattering by dense dislocations as well as lattice distortion. Finally, a high *zT* value of 1.1 at 400 K is obtained in the n‐type LPHD Bi_2_(Te,Se)_3_ alloys. These results not only demonstrate a simple and energy‐saving technique for synthesizing high‐performance n‐type Bi_2_Te_3_‐based materials, but also emphasize the significance of liquid eutectic phase in tuning materials' microstructures.

## 2 Results and Discussion

**Figure 1:**
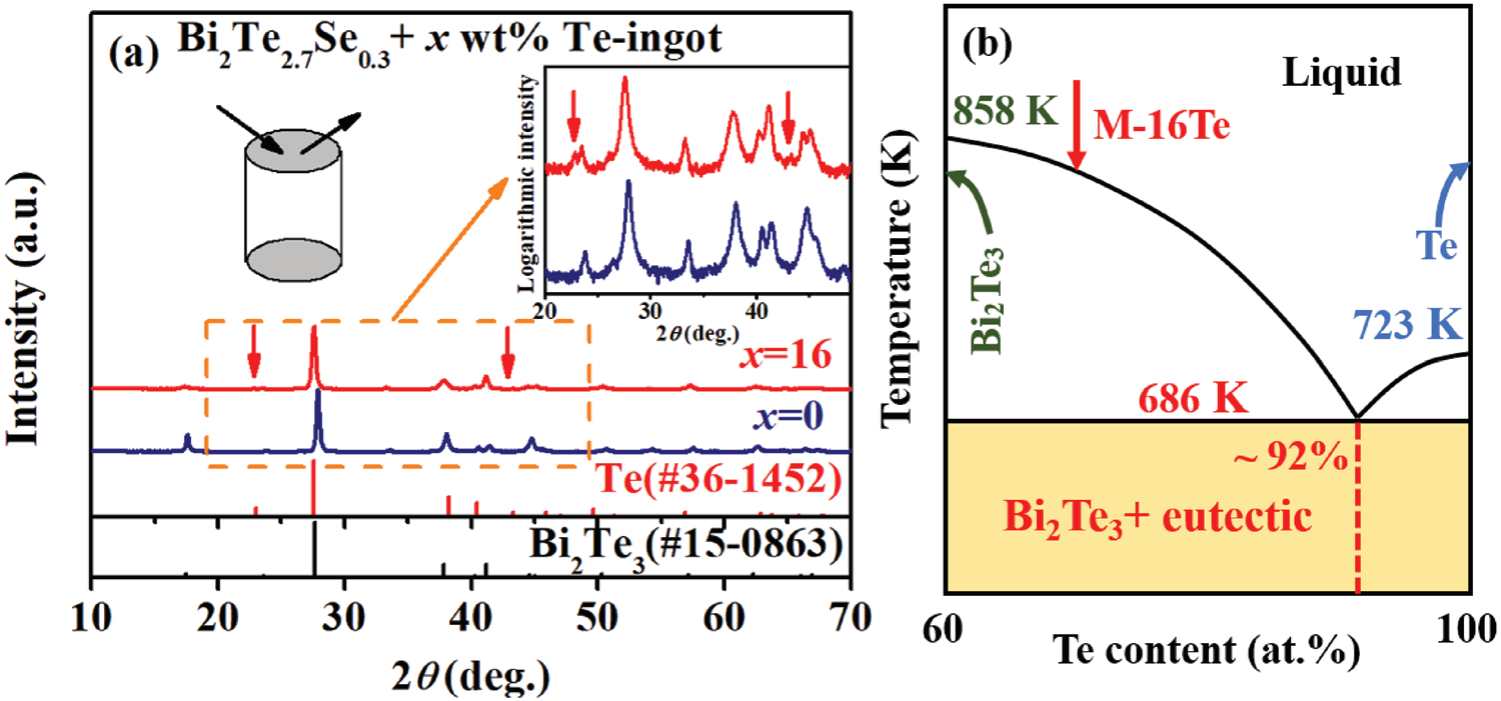
a) XRD patterns of Bi_2_Te_2.7_Se_0.3_ + *x* wt% Te melted ingot. Inset is the enlarged XRD pattern with a logarithmic intensity axis. The red arrows indicate the peaks from elemental Te. b) Phase diagram of Bi_2_Te_3_–Te system with an eutectic composition at ≈92 at% Te.[qv: 4e,16] The red arrow indicates the nominal composition of Bi_2_Te_2.7_Se_0.3_ + 16 wt% Te melted ingot.

The X‐ray diffraction (XRD) patterns in **Figure** **1**a indicate that the Bi_2_Te_2.7_Se_0.3_ melted ingot (named as M‐0Te) has a pure rhombohedral *R3m* phase, while the Bi_2_Te_2.7_Se_0.3_ + 16 wt% Te‐melted ingot (named as M‐16Te) has a little Te second phase remaining in the Bi_2_Te_3_ matrix. The Te peaks (indicated by the red arrows) are hard to be recognized from the normal XRD patterns but can be clearly identified in the logarithmic XRD intensity versus 2θ patterns.

**Figure 2:**
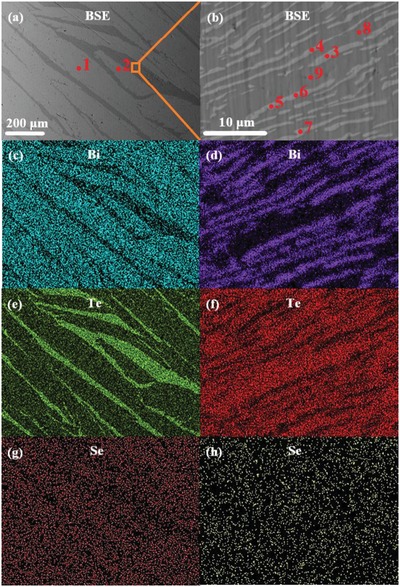
a) SEM backscattered electron images for Bi_2_Te_2.7_Se_0.3_ + 16 wt% Te melted ingot. b) Enlarged view of boxed region in panel (a). c) Bi, e) Te, and g) Se elemental distribution in panel (a). d) Bi, f) Te, and h) Se elemental distribution in panel (b).

The scanning electron microscopy (SEM) backscattered electron (BSE) image (**Figure** **2**a) and its corresponding elemental distribution mapping results (see Figure 2c,e,g) also reveal the existence of Te‐rich phase in the M‐16Te. The brighter phase in Figure 2a is roughly identified as Bi_2_Te_2.55_Se_0.33_ by energy‐dispersive spectrometer (EDS; Table S1, Supporting Information) and then corrected to be Bi_2_Te_2.66_Se_0.26_ by electron probe microanalysis (EPMA; Table S2, Supporting Information), while the darker phase (with more Te) in Figure 2a exhibits a lamellar structure composed of two phases with different Te contents, as shown in Figure 2b and Figure S1 (Supporting Information). Further analysis on the lamellar structure (Figure 2d,f,h; Tables S1 and S2, Supporting Information) indicates that the darker region is pure Te, while the composition of brighter region is variable because the grain size is too small (≤1 µm) to be explicitly examined by EDS and EPMA. Based on the Bi_2_Te_3_–Te phase diagram in Figure 1b,[qv: 4e,16] this lamellar structure might be the eutectic phase of Bi_2_Te_3_–Te. To verify it, a differential scanning calorimetry (DSC)– thermogravimetric analysis (TGA) test is performed on the M‐16Te, and a distinct endothermic peak at 692.3 K is detected (see Figure S3 in the Supporting Information), very close to the eutectic temperature (686 K) in Figure 1b. Therefore, the microstructure of M‐16Te is concluded to be a mixture of Bi_2_Te_2.66_Se_0.26_ alloys and eutectic phase of Bi_2_(Te,Se)_3_–Te. Undoubtedly, the Bi_2_(Te,Se)_3_–Te eutectic phase would also exist in our other Bi_2_Te_2.7_Se_0.3_ + *x* wt% Te‐melted ingots (*x* from 1 to 32) according to Figure 1b.

**Figure 3:**
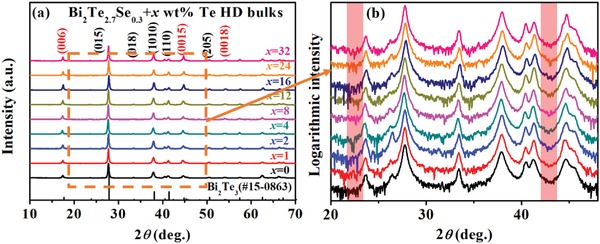
a) In‐plane XRD patterns of the HD‐0Te and LPHD‐*x*Te bulk samples. b) Enlarged XRD patterns with a logarithmic intensity axis. Compared to the inset in Figure 1a, no peaks from elemental Te are detected in the red box range.

Since the HD temperature is above the eutectic point, the deformation of Bi_2_(Te,Se)_3_ bulk is accompanied by the extrusion of liquid eutectic phase. Hence, this procedure is named as liquid‐phase hot deformation and the picture of extruded eutectic phase is shown in Figure S3 (Supporting Information). For simplicity, the HD Bi_2_Te_2.7_Se_0.3_ sample is named as HD‐0Te and other HD Bi_2_Te_2.7_Se_0.3_ + *x* wt% Te (*x* ≥ 1) samples are named as LPHD‐*x*Te, respectively. The XRD patterns in **Figure** **3**a,b show that all HD‐0Te or LPHD‐*x*Te samples are pure Bi_2_Te_3_ phase without any detectable Te second phase remaining. The compositional homogeneity in all samples is confirmed by EDS mapping and a typical result of LPHD‐16Te is shown in Figure S4 (Supporting Information). The real composition of LPHD‐16Te is identified as Bi_2_Te_2.70_Se_0.27_ by EPMA, which has a slightly higher Te and Se contents compared to the Bi_2_Te_2.66_Se_0.26_ melted precursor ingot.

**Figure 4:**
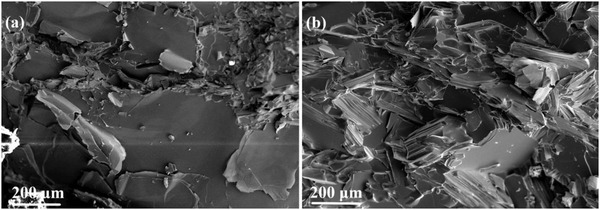
SEM images of the cross sections parallel to the pressing direction for the a) HD‐0Te sample and b) LPHD‐16Te sample.

To evaluate the texture degree, orientation factor *F* of (000*l*) plane was calculated using the Lotgering method,^17^ and the results are summarized in **Table** **1**. It could be seen that with the increase of *x* in LPHD‐*x*Te, *F* first increases from 0.09 to 0.20 and then slightly decreases to 0.17, indicating an initially enhanced and then weakened texture in the LPHD samples (the nonmonotonic variation of *F* will be discussed later). A comparison of SEM microstructure between HD‐0Te and LPHD‐16Te is also presented in **Figure** **4**; the coarse grains with random distribution are exhibited in HD‐0Te, while the grains are refined and highly oriented in the LPHD‐16Te, consistent with the *F* results.

**Table 1:** The orientation factor *F* of (000*l*) plane for the HD‐0Te and LPHD‐*x*Te bulk samples

*x* content	0	1	2	4	8	12	16	24	32
*F* value	0.09	0.12	0.14	0.17	0.17	0.18	0.20	0.17	0.17

**Figure 5:**
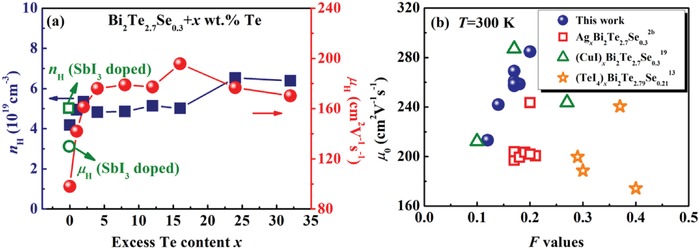
a) Room temperature carrier concentration and mobility as a function of excess Te content *x* in the HD‐0Te and LPHD‐*x*Te samples. The green spots are the HD Bi_2_Te_2.7_Se_0.3_ sample with SbI_3_ doping. b) Nondegenerated mobility *µ*_0_ as a function of orientation factor *F* in LPHD Bi_2_Te_2.7_Se_0.3_ samples and other reported HD Bi_2_Te_2.7_Se_0.3_ and HD Bi_2_Te_2.79_Se_0.21_ samples.[qv: 2b,13,19]

An increase of *n*_H_ from 4.1 × 10^19^ to 5.0–6.4 × 10^19^ cm^−3^ after LPHD is shown in **Figure** **5**a, indicating that excess Te acts as electron donor during the LPHD process, similar to the case in the n‐type Te‐doped Bi_2_Te_2.4_Se_0.6_ alloys.^18^ The compositional variation in the LPHD‐16Te sample (from Bi_2_Te_2.66_Se_0.26_ to Bi_2_Te_2.70_Se_0.27_) indicates that a small number of Te and Se atoms in the liquid eutectic phase may diffuse into the Bi_2_(Te,Se)_3_ grains during LPHD and provide electrons (in the formation of ${\mathrm{Te}}_{\mathrm{Bi}}^{•}$ antisite defects). Nevertheless, when increasing *x* from 1 to 32, *n*_H_ has little change, which may be caused by the relatively small equilibrium concentration of ${\mathrm{Te}}_{\mathrm{Bi}}^{•}$ in n‐type Bi_2_(Te,Se)_3_ alloys. The variation of room temperature carrier mobility *µ*_H_ with *x* is also presented in Figure 5a. As one can see, *µ*_H_ first increases and then decreases with *x*, reaching *µ*_H_ ≈ 196 cm^−2^ V^−1^ s^−1^ for the LPHD‐16Te sample (the nonmonotonic variation of *µ*_H_ will be discussed later). Low‐temperature Hall measurement (Figure S5a, Supporting Information) shows that the intrinsic conduction near room temperature is obvious in the HD‐0Te sample, which will result in an incorrectly calculated *µ*_H_. Even so, the enhanced *µ*_H_ by LPHD could be verified from the *µ*_H_ data in the extrinsic region (*T* < 150 K), consistent with the *F* results in Table 1. To eliminate the influence of intrinsic conduction and make a better comparison between the normal HD sample and LPHD samples, a heavy SbI_3_‐doped Bi_2_Te_2.7_Se_0.3_ HD sample was also prepared, and its carrier transport properties are labeled in Figure 5a. Apparently, the *µ*_H_ of LPHD samples is still higher than the HD sample, demonstrating the importance of liquid eutectic phase for enhancing *µ*_H_ during the LPHD process.

A comparison of Hall mobility *µ*_H_ and orientation factor *F* between LPHD Bi_2_Te_2.7_Se_0.3_ samples and other reported HD Bi_2_Te_2.7_Se_0.3_ or Bi_2_Te_2.79_Se_0.21_ samples are summarized in Figure S5b (Supporting Information). In the single parabolic band (SPB) model, *µ*_H_ is readily influenced by the reduced Fermi energy η according to the following equation^20^

(1) $$\mu_{H}=\mu_{0}\frac{\left(1/2+2\lambda \right)F_{2\lambda -1/2}}{\left(1+\lambda \right)F_{\lambda }}$$

(2) $$F_{j}\left(\eta \right)=\int_{0}^{\infty }\frac{x^{j}}{1+e^{x-\eta }}dx$$

where λ is the scattering parameter and is equal to 0 for acoustic phonon scattering, *F_j_*(η) is the Fermi integral of order *j*, *µ*_0_ is the nondegenerate mobility and related to the carrier relation time τ_0_ (affected by the carrier scattering), transport effective mass $m_{I}^{*}$, and electron charge *e* as following^20^

(3) $$\mu_{0}=e\tau_{0}/m_{I}^{*}$$

In the SPB model, η could be calculated by α according to the following equation^20^

(4) $$\alpha =\pm \frac{k_{B}}{e}\left(\frac{\left(2+\lambda \right)F_{\lambda +1}\left(\eta \right)}{\left(1+\lambda \right)F_{\lambda }\left(\eta \right)}-\eta \right)$$

In order to exclude the effect of η on carrier mobility, the nondegenerate mobility *µ*_0_ of all samples in Figure S5b (Supporting Information) was calculated according to Equations (1)–(3) with the assumption of acoustic phonon scattering dominating, and the results are presented in Figure 5b. Compared to the routine HD samples,[qv: 2b,19] our LPHD samples have higher *µ*_0_ at a fixed *F*. Particularly, although the HD Bi_2_Te_2.79_Se_0.21_ samples^13^ have weaker alloy scattering and larger *F* values, their *µ*_0_ is still less than that of the LPHD Bi_2_Te_2.7_Se_0.3_ alloys. It should be noted that at a constant *F* value, *µ*_0_ is only related to τ_0_ and $m_{I}^{*}$, which is described in Equation (3). Since our LPHD samples have a similar composition with other reported HD samples, the variation of $m_{I}^{*}$ among these samples could be ignored. Therefore, the remarkably enhanced *µ*_0_ by LPHD should arise from the increased carrier relation time τ_0_, which indicates a reduced carrier scattering in the LPHD samples.

**Figure 6:**
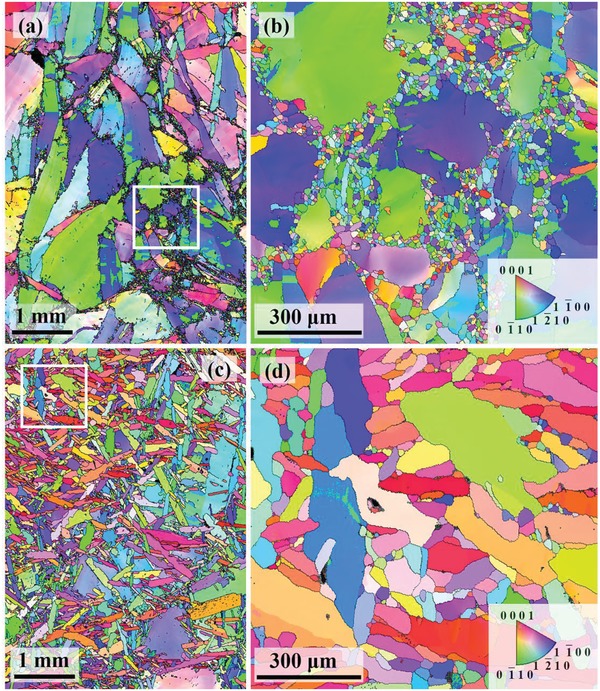
Orientation imaging microscopy maps for the a) HD‐0Te sample and c) LPHD‐16Te sample. b) The enlarged view of the box region in panel (a), and d) the enlarged view of box region in panel (c).

To verify this conjecture, further microstructural investigations on our HD‐0Te and LPHD‐16Te samples was carried out by electron backscattered diffraction (EBSD). The results are shown in **Figure** **6**. Figure 6a shows that the HD‐0Te sample has the coarse grains with numerous fine grains in the vicinity of large grains. Figure 6b is a zoom‐up of the box region in Figure 6a, confirming that the dense dots around the coarse grain boundaries are fine equiaxed grains rather than other defects or holes. A highly inhomogeneous distribution of grain size in the HD‐0Te sample is presented in Figure S6a (Supporting Information); the coarsest grain size reaches ≈1 mm, while the finest grain size is only ≈4 µm and could be smaller due to the limited scanning step size of 2.5 µm. The coarse grains are considered to be generated at the melting–cooling stage and be elongated during hot deformation, while the fine equiaxed grains should be the hot deformation‐induced dynamic recrystallized grains by comparing the typical dynamic recrystallization microstructures in the plastic deformed metals.^21^

**Figure 7:**
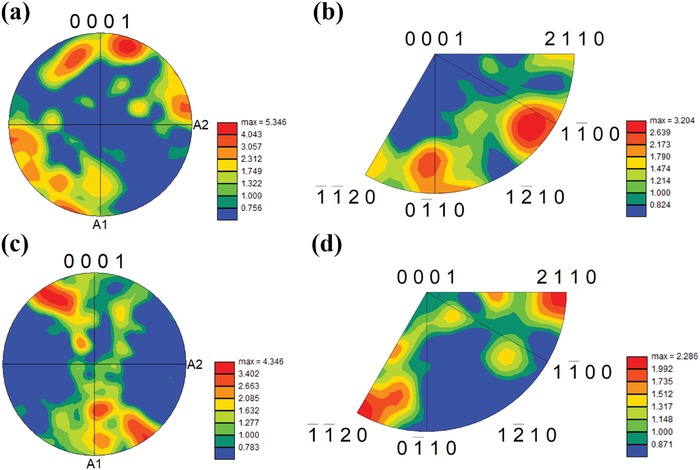
a) Pole figure of (0001) plane and b) inverse pole figure for the HD‐0Te sample. c) Pole figure of (0001) plane and d) inverse pole figure for the LPHD‐16Te sample.

**Figure 8:**
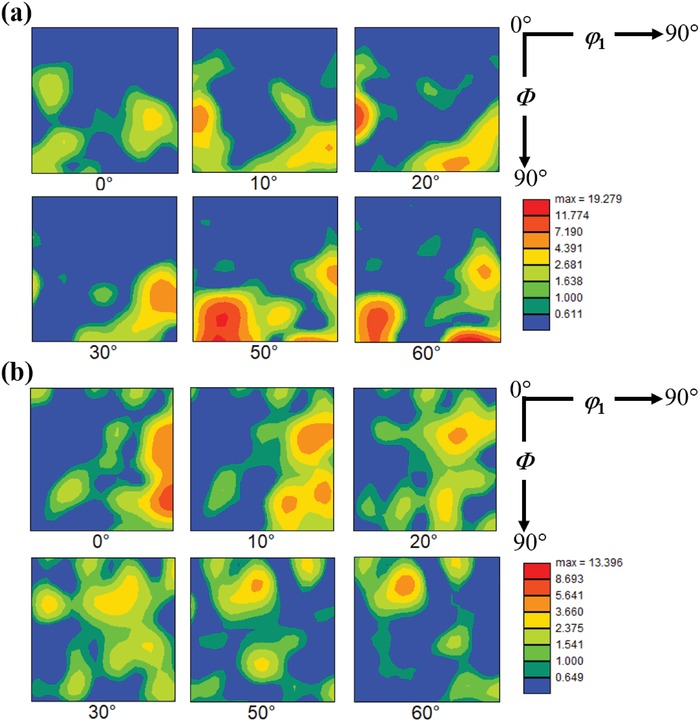
Partial ODF patterns for the a) HD‐0Te sample and b) LPHD‐16Te sample.

In contrast, the LPHD‐16Te sample has more bar‐shaped grains with a relatively homogeneous distribution of grain size, as shown in Figure 6c and Figure S6 (Supporting Information). Note that, compared to the HD‐0Te sample, the number of recrystallized grains is significantly decreased in the LPHD sample (see Figure 6d and Figure S6 in the Supporting Information) combined with a reduction of total boundary length from 2.15 m (Figure 6a) to 1.58 m (Figure 6c) in an area of 4 mm by 6 mm, which is beneficial for the reduction of carrier scattering and enhancement of *µ*_H_. The improved *µ*_H_ also results from strongly texturing during LPHD. In Figure 6, a larger regime of red color represents a stronger (000*l*) preferred orientation, and it is easy to find that the LPHD sample has more enhanced (000*l*) texture. This is further demonstrated by the pole figure (POF) and inverse pole figure (IPF) results in **Figure** **7**, since a higher polar density in the center of (0001) POF and near the (0001) point in IPF indicates a stronger (000*l*) texture.^22^ Furthermore, enhanced texture in the LPHD sample is also double‐checked by part of the orientation distribution function (ODF) results in **Figure** **8**. For Bi_2_Te_3_ alloys, more highlighted regions located at the top of ODF sections (Φ = 0) indicate stronger texture along the (000*l*) direction. More detailed ODF analysis can be found in Figure S7 (Supporting Information).

As mentioned above, both the diminution of recrystallized grains and enhancement of texture during the LPHD process are beneficial for the increase in *µ*_H_. The former could be well illustrated based on the dynamic recrystallization theory.[qv: 21b,23] In a normal hot deformation process, dynamic recrystallization takes place during straining as long as the temperature is heated above half of the melting point of samples. The recrystallized grains are produced by nucleation and growth of crystallites in the vicinity of grain boundaries and the driving force is the stored energy induced by the plastic deformation. Hence, HD‐induced recrystallized grains could be readily found in the HD‐0Te sample (see Figure 6a,b) and these grains also exist in normal hot‐deformed Bi_2_Te_3_ materials.[qv: 4c] However, during the LPHD process, most of the grains are surrounded by liquid eutectic phase until the liquid is completely extruded, resulting in a decreased nucleation sites at grain boundaries. In addition, the stress induced by deformation is timely released by the extrusion of liquid, hence the stored energy in grains maintains on a relatively low level, further inhibiting nucleation. Therefore, the dynamic crystallization is well suppressed in the LPHD‐16Te sample (see Figure 6c,d).

The enhanced texture during LPHD is also related to the eutectic phase. For HD Bi_2_Te_3_ alloys, grain rotation along the basal plane is demonstrated to be the main mechanism for texturing.^13^, ^24^ Coarse grains are quite difficult to rotate and hence the texture is relatively low in the HD‐0Te sample. Comparatively, for the LPHD sample, grains are refined by the constraint of eutectic phase at the melting–cooling stage and the rotation resistance at the HD stage is also reduced by the wetting of liquid phase, both contributing to an easier grain rotation and stronger texture during the LPHD process. Compared to the normal HD process, in which *µ*_H_ could be deteriorated by recrystallization, the LPHD procedure can simultaneously enhance the texture and suppress the dynamic recrystallization, resulting in a much higher carrier mobility in the LPHD samples (see Figure 5b).

**Figure 9:**
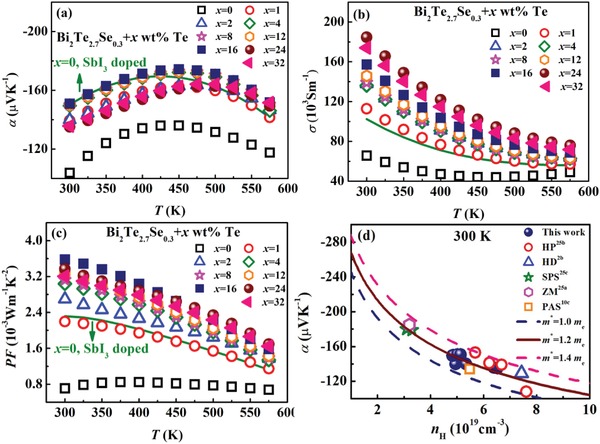
Temperature dependences of a) Seebeck coefficient, b) electrical conductivity, and c) power factor for the HD‐0Te and LPHD‐*x*Te samples. The green curve is the HD Bi_2_Te_2.7_Se_0.3_ sample with SbI_3_ doping. d) Room‐temperature Pisarenko plots of the LPHD‐*x*Te samples and SbI_3_‐dope HD sample with other Bi_2_Te_2.7_Se_0.3_ literature data.[qv: 2b,10c,25]

The electrical properties of HD‐0Te and LPHD‐*x*Te samples are displayed in **Figure** **9**. The remarkable intrinsic conduction near room temperature results in a relatively low α in the HD‐0Te sample (see Figure 9a). For the SbI_3_‐doped HD and LPHD samples, bipolar effect is suppressed by the increased *n*_H_ and hence α is improved. In addition, the slight fluctuation of α with *x* corresponds well to the variation of *n*_H_ in Figure 5a, suggesting that the different Te contents in LPHD‐*x*Te samples and SbI_3_‐doped HD sample do not cause noticeable change in effective mass m*. This is verified by the Pisarenko plots in Figure 9d, and the m* for LPHD and SbI_3_‐doped HD samples is calculated to be ≈1.2 *m*_e_ at 300 K, consistent well with the results reported by others.[qv: 2b,10c,25]

Attributed to the enhanced *µ*_H_ at *x* ≤ 16 or slightly increased *n*_H_ at *x* ≥ 24 (see Figure 5a), σ roughly increases with *x* in the LPHD‐*x*Te samples, as shown in Figure 9b. By tracing the degree of recrystallization and evolution of grains in LPHD samples, the nonmonotonic variation of *µ*_H_ with *x* (in Figure 5a) can be interpreted as follows: in an ideal condition, the number of recrystallized grains in LPHD samples should continuously decrease with the increase of eutectic phase. However, in a certain HD process, during which the HD temperature, HD pressure and HD degree of sample are all constant, the total number of produced recrystallized grains in the matrix is limited. With the increase of eutectic phase content during LPHD, less recrystallized grains are reduced in the matrix, thus leading to less improvement of *µ*_H_. On the other hand, as mentioned above, during the melting–cooling stage, the grains are refined by the constraint of eutectic phase, hence grain size should monotonously decrease with the increase of eutectic content. Although eutectic phase is beneficial for texturing through facilitating grain rotation, it also causes significant reduction of grain size and increase of grain boundaries, which could lead to weaker texture^26^ (see the *F* values in Table 1) and enhanced carrier scattering. As a result, the gain from suppressed dynamic recrystallization is offset by the degraded texture and increased grain boundaries, leading to a compromise at *x* = 16 with a maximal *µ*_H_ ≈ 196 cm^−2^ V^−1^ s^−1^. The PFs for all samples are plotted in Figure 9d. Compared to the HD‐0Te sample, PF in LPHD samples is significantly boosted by the simultaneous optimization of α and σ. The maximal PF ≈ 3.6 × 10^−3^ W m^−1^ K^−2^ is obtained at *x* = 16, about 60% increment over the HD SbI_3_‐doped sample.

**Figure 10:**
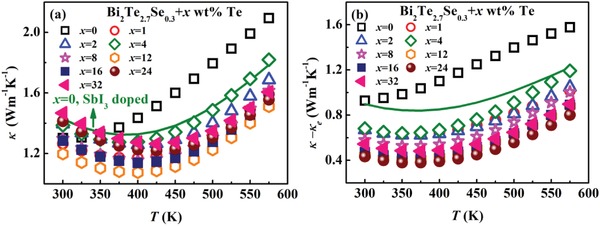
Temperature dependences of in‐plane a) thermal conductivity and b) lattice thermal conductivity for the HD‐0Te and LPHD‐*x*Te samples. The green curve is the HD Bi_2_Te_2.7_Se_0.3_ sample with SbI_3_ doping.

The total thermal conductivity κ of HD‐0Te and LPHD‐*x*Te samples is presented in **Figure** **10**a. Due to the increased *n*_H_ by Te doping, bipolar conduction is suppressed at room temperature in the LPHD samples. The electronic thermal conductivity κ_e_ of all samples is calculated according to κ_e_ = *LσT*, where *L* is the Lorenz number and estimated by the SPB model. The results are shown in Figure S8 (Supporting Information). Due to the enhanced σ, κ_e_ roughly increases with *x* in the LPHD‐*x*Te samples. Figure 10b shows the temperature dependence of lattice thermal conductivity κ_L_ calculated by κ – κ_e_. An obvious decrease of room temperature κ_L_ is observed in the LPHD samples. In particular, the minimal room temperature κ_L_ ≈ 0.43 W m^−1^ K^−1^ is achieved at *x* = 24, almost 50% reduction than that of HD SbI_3_‐doped sample. As mentioned above, compared to the HD‐0Te sample, although coarse grains are refined in the LPHD‐16Te sample, the number of fine recrystallized grains is also remarkably decreased, contributing to a reduced total grain boundary length in the LPHD‐16Te sample. In this case, low‐frequency phonon scattering by grain boundary should be weakened in the LPHD sample. Therefore, the reduction of κ_L_ by LPHD must come from other reasons.

**Figure 11:**
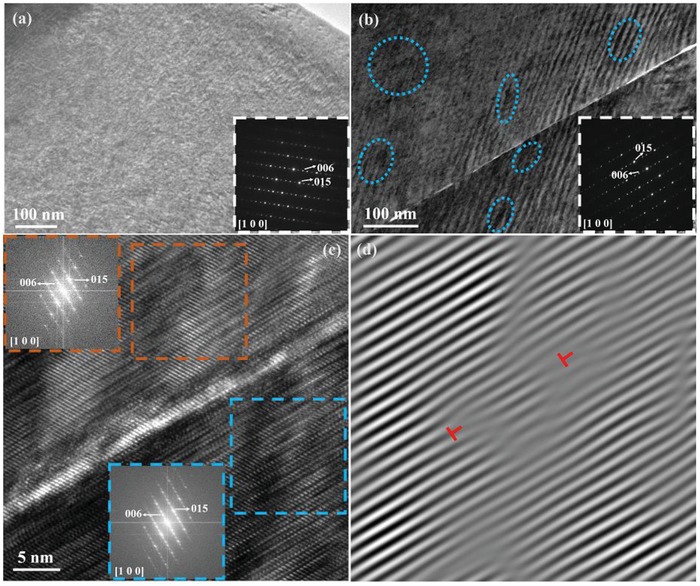
a) Low‐magnification TEM image of the HD‐0Te sample with its electron diffraction pattern (inset). b) Low‐magnification TEM image of LPHD‐16Te sample with its electron diffraction pattern (inset). c) HRTEM image from panel (b) with two FFT images of different positions. d) Inverse FFT image of the blue box in panel (c) obtained from (006) reflection.

In order to clearly illustrate the influence of LPHD on the κ_L_ in Bi_2_Te_2.7_Se_0.3_ alloys, further microstructure observation is conducted by a transmission electron microscope (TEM), and the results are shown in **Figure** **11**. It can be clearly seen that compared to the HD‐0Te sample (Figure 11a), the LPHD‐16Te sample possesses more strain‐field domains (Figure 11b, marked with blue dashed curves), which are ascribed to dislocation effect in previous report.^27^ The bright straight line in Figure 11b is identified as a low‐angle grain boundary by the high‐resolution TEM (HRTEM) image in Figure 11c, in which two quite similar fast Fourier transform (FFT) images of adjacent grains are also displayed. More grain boundary images observed along different TEM zone axis are presented in Figure S9 (Supporting Information). To further investigate the strain‐field domains in the LPHD sample, inverse Fourier transformation is performed on the blue box region in Figure 11c, and the obtained inverse FFT (IFFT) pictures along (006) and (015) reflections are shown in Figure 11d and Figure S10 (Supporting Information), respectively. Large‐scale lattice distortion and dense dislocations (marked by red symbol) can be readily found in the IFFT, which should play important roles in scattering high‐ and medium‐frequency phonons. For HD‐0Te sample, a large number of defects are first induced by plastic deformation and then readily diminished by recrystallization, hence resulting in a relatively high κ_L_. In contrast, for LPHD sample, although the deformation‐induced dislocations and lattice distortion are considerably decreased by the extrusion of liquid eutectic phase, these defects can always remain in the matrix due to the remarkable suppression of recrystallization. As a result, strong scattering of phonons is realized in the LPHD samples, contributing to a remarkable reduction of κ_L_.

**Figure 12:**
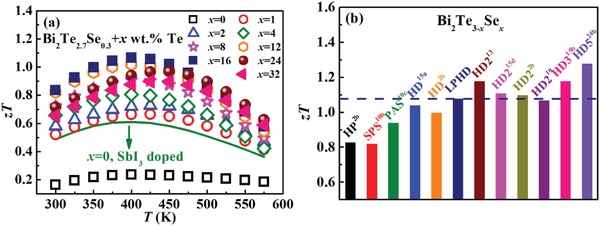
a) Temperature dependences of *zT* for the HD‐0Te sample and LPHD‐*x*Te samples. The green curve is the HD Bi_2_Te_2.7_Se_0.3_ sample with SbI_3_ doping. b) Maximum *zT* values for the LPHD sample and other reported Bi_2_Te_3−_ *_x_*Se*_x_* alloys (0.21 ≤ *x* ≤ 0.8).[qv: 2b,10b,c,13,15a,b,d,19,24b]

The dimensionless figures of merit, *zTs*, for all HD and LPHD Bi_2_Te_2.7_Se_0.3_ samples are presented in **Figure** **12**a. With varying *x* from 0 to 32, the maximum *zT* first increases and then decreases. The highest *zT* value around 1.1 at 400 K is obtained for the LPHD‐16Te sample, about 75% increment over the HD SbI_3_‐doped one, even though both of them have the same *n*_H_ ≈ 5.0 × 10^19^ cm^−3^. As aforementioned, liquid‐phase hot deformation could effectively suppress the dynamic recrystallization and enhance the texture, both resulting in a remarkable enhancement of carrier mobility. Meanwhile, dense dislocations and lattice distortion are also introduced into the LPHD samples, contributing to an obvious reduction of κ_L_ and further enhancement of *zT*. A comparison of maximum *zT* values among LPHD‐16Te sample and other reported Bi_2_Te_3−_ *_x_*Se*_x_* alloys[qv: 2b,10b,c,13,15a,b,d,19,24b] is presented in Figure 12b. Comparing with the normal powder metallurgical process and single HD process, higher *zT* could be obtained by LPHD technique. Although *zTs* in some multiple HD alloys are slightly higher than *zT* in the LPHD sample, the process flow of one‐step LPHD technique is much shorter, which makes it a more efficient and energy‐saving route for large‐scale production of superior n‐type Bi_2_(Te,Se)_3_ materials.

## 3 Conclusion

Here, a liquid‐phase hot deformation procedure is successfully performed to enhance the thermoelectric performance of n‐type Bi_2_(Te,Se)_3_ alloys. The Te‐rich eutectic phase is introduced into the Bi_2_(Te,Se)_3_ ingot at the melting stage and plays important roles in subsequent hot deformation stage. First, the nucleation sites and stored energy for dynamic recrystallization are both reduced due to the wetting and extrusion of liquid eutectic phase, leading to a decrease of recrystallized grains and hence weakened carrier scattering, which is beneficial for an enhanced carrier mobility. Second, grain rotation along the (000*l*) plane becomes easier with the help of liquid phase, contributing to a stronger texture and further boosted *µ*_H_. Third, lattice thermal conductivity is also simultaneously decreased by the dense dislocations and lattice distortion introduced during LPHD. All these effects contribute to a high *zT* ≈ 1.1 at 400 K in the n‐type LPHD Bi_2_(Te,Se)_3_ alloys. This work demonstrates a simple technique for achieving superior n‐type Bi_2_Te_3_‐based materials, which is much more efficient and energy saving compared to the multiple HD process.

## 4 Experimental Section

Bi (5 N), Te (5 N), Se (5 N) element chunks were weighted according to the stoichiometric Bi_2_Te_2.7_Se_0.3_ + *x* wt% Te (*x* = 0, 1, 2, 4, 8, 12, 16, 24, 32) and sealed into φ 12.7 mm quartz tubes evacuated to 10^−3^ Pa. The mixtures were subsequently melted at 1173 K for 10 h in a furnace and rocked every 2 h to ensure the composition homogeneity. The quartz tubes were sufficiently rocked before taken out from the furnace and then cooled in the air. The conical bottom part of the obtained ingot was cut off and the remained φ 12.7 mm cylindrical ingot was directly hot‐deformed in a larger φ 20 mm graphite die at 773 K for 30 min with 80 MPa uniaxial pressure. The HD direction was parallel to the axial direction of the cylinder. Finally, disk‐shaped HD samples with high densities (>97% of theoretical density) were required.

The phase structures of all samples were evaluated by the XRD on a Rigaku D/MAX‐2550P diffractometer. The freshly fractured surfaces of bulk samples and the microstructure of melted ingots were observed by SEM (Hitachi S‐3700N) equipped with an EDS. The chemical compositions were investigated by EDS and checked by EPMA (JEOL, JXA‐8100) using a wavelength dispersive spectroscope (WDS). The TEM observation was performed on FEI TF20 microscopes, and the TEM samples were prepared by the dual‐beam FIB equipment (Quanta 3D FEG, FEI). The samples for EBSD measurement were first grinded and then mechanically polished by diamond paste (3 in roughness) for about 15 min and finally fine polished by oxide polishing suspension solution for 30 min with an applied load of 60 N. The EBSD analysis was performed on a dual‐beam focused ion beam (FIB, Helios NanoLab 600i, FEI) using a Hikari S/N 1040 camera (TSL/EDAX). EBSD data acquisition and analysis were performed by the OIM Data Collection software and OIM Analysis 7 software, respectively. The DSC and TGA analyses were simultaneously carried out on a TA Instrument SDT Q600 thermal analyzer with a heating rate of 10 K min^−1^ under Ar atmosphere.

The electrical conductivity σ and Seebeck coefficient α were measured on a commercial Linseis LSR‐3 system using a differential voltage/temperature technique and a DC four‐probe method. The thermal conductivity κ was calculated by using κ = *DρC*_P_, where ρ is the density estimated by an ordinary dimension‐and‐weight method, *C*_P_ is the specific heat calculated by the Dulong–Petit law, and *D* is the thermal diffusivity measured by a laser flash method on a Netzsch LFA 467 instrument with a Pyroceram standard. The samples for *D* measurement were prepared using the same method with Xie et al.^28^ The low‐temperature electrical conductivity σ and Hall coefficient *R*_H_ from 10 to 300 K were measured on a Mini Cryogen Free Measurement System (Cryogenic Limited, UK). Then the Hall carrier concentration *n*_H_ and Hall mobility *µ*_H_ were calculated via *n*_H_ = 1/*eR*_H_ and *µ*_H_ = *σR*_H_, respectively. In particular, all thermoelectric properties were measured along the in‐plane direction of samples.

## Conflict of Interest

The authors declare no conflict of interest.

## Supporting information

Supplementary
