# Supplementary material for: Liquid‐Phase Hot Deformation to Enhance Thermoelectric Performance of n‐type Bismuth‐Telluride‐Based Solid Solutions
Source: Adv Sci (Weinh). 2019 Sep 14;6(21):1901702. doi: 10.1002/advs.201901702 (PMC6839625; doi:10.1002/advs.201901702)
Supplement: Supplementary file 1 — Supplementary [file ADVS-6-1901702-s001.pdf]

Copyright WILEY-VCH Verlag GmbH & Co. KGaA, 69469 Weinheim, Germany, 2019.

ADVANCED SCIENCE
Open Access

Supporting Information

for *Adv. Sci.,* DOI: 10.1002/advs.201901702

Liquid-Phase Hot Deformation to Enhance Thermoelectric Performance of n-type Bismuth-Telluride-Based Solid Solutions

Yehao Wu, Yuan Yu, Qi Zhang, Tiejun Zhu, Renshuang Zhai, and Xinbing Zhao**

Copyright WILEY-VCH Verlag GmbH & Co. KGaA, 69469 Weinheim, Germany, 2019.

Supporting Information

Liquid-Phase Hot Deformation to Enhance Thermoelectric Performance of *n*-type Bismuth-Telluride Based Solid Solutions

Yehao Wu, Yuan Yu, Qi Zhang, Tiejun Zhu, Renshuang Zhai, and Xinbing Zhao**

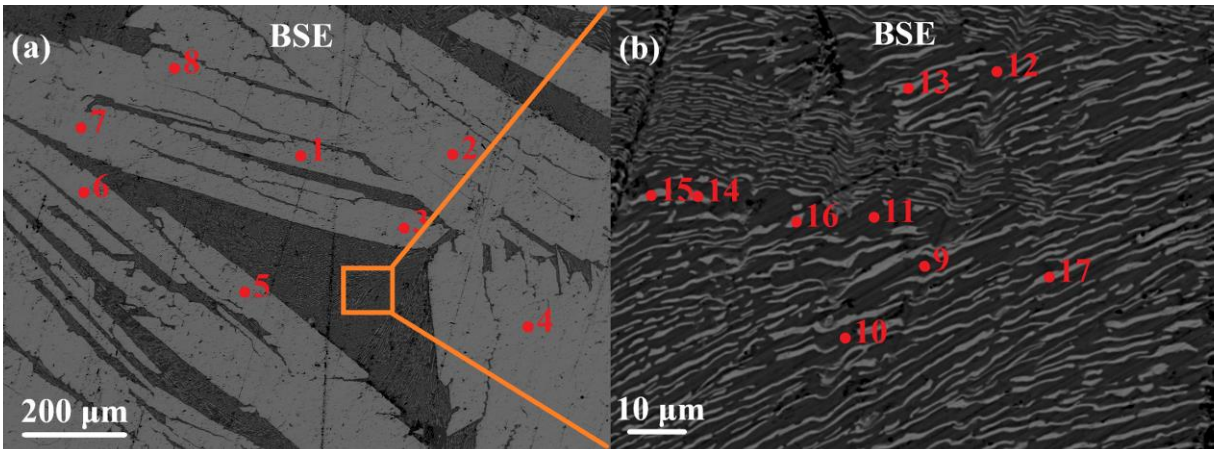

Figure S1. a) EMPA backscattered electron (BSE) images for $Bi_2Te_{2.7}Se_{0.3}$ + 16 wt.% Te melted ingot. b) Enlarged view of boxed region in (a).

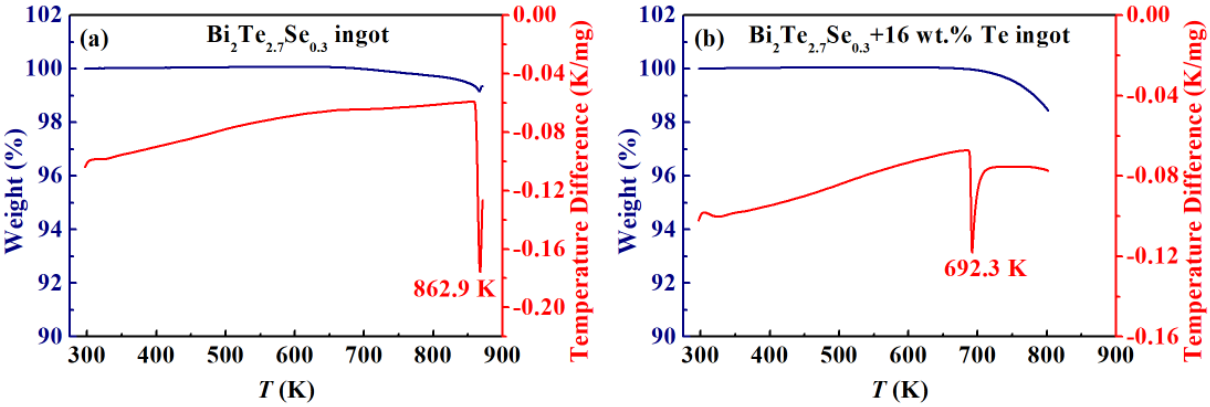

Figure S2. DSC and TGA curves for a) $Bi_2Te_{2.7}Se_{0.3}$ and b) $Bi_2Te_{2.7}Se_{0.3}$ + 16 wt.% Te melted ingots. The testing temperature for $Bi_2Te_{2.7}Se_{0.3}$ + 16 wt.% Te melted ingot is only up to 802 K due to the volatilization of Te.

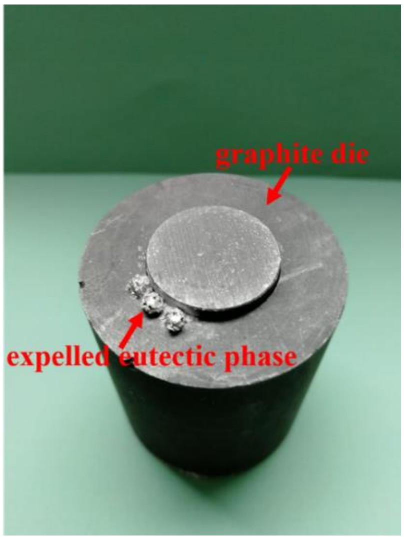

Figure S3. Picture after liquid-phase hot deformation for the $Bi_2Te_{2.7}Se_{0.3}$ + 16 wt.% Te ingot.

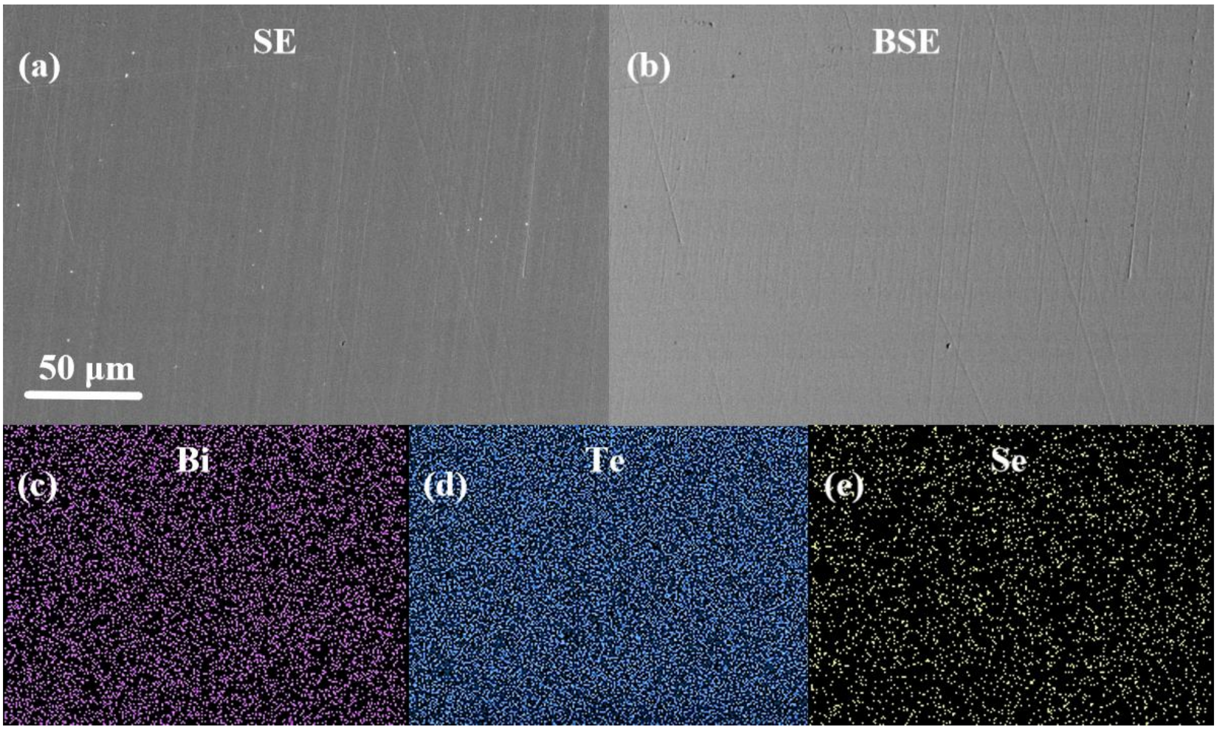

Figure S4. a) Second electron and b) backscattered electron images for LPHD-16Te sample. c) Bi, d) Se and e) Te elemental distribution in (a).

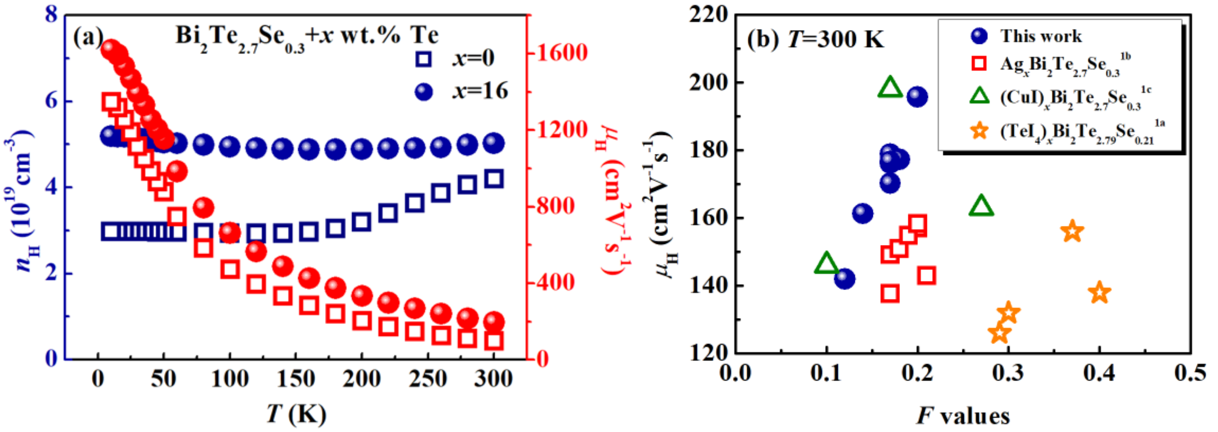

Figure S5. a) Temperature dependences of carrier concentration and mobility for the HD-0Te and LPHD-16Te samples (from 10 K to 300 K). b) Room temperature Hall mobility μ_H as a function of orientation factor F for our LPHD $Bi_2Te_{2.7}Se_{0.3}$ samples and other reported HD $Bi_2Te_{2.7}Se_{0.3}$ and HD $Bi_2Te_{2.79}Se_{0.21}$ samples.[1]

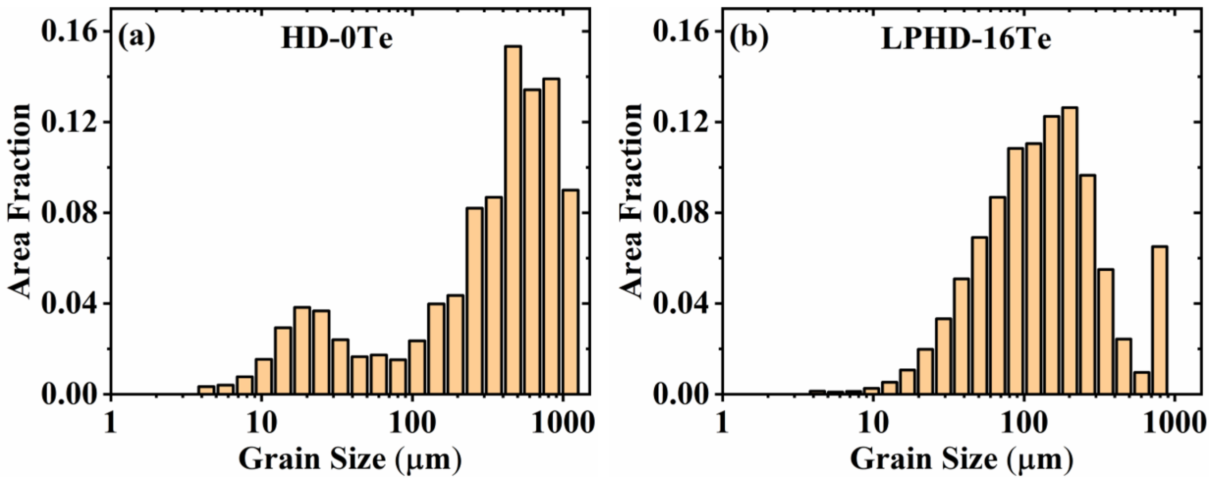

Figure S6. The statistic of average grain size in the a) HD-0Te sample and b) LPHD-16Te sample.

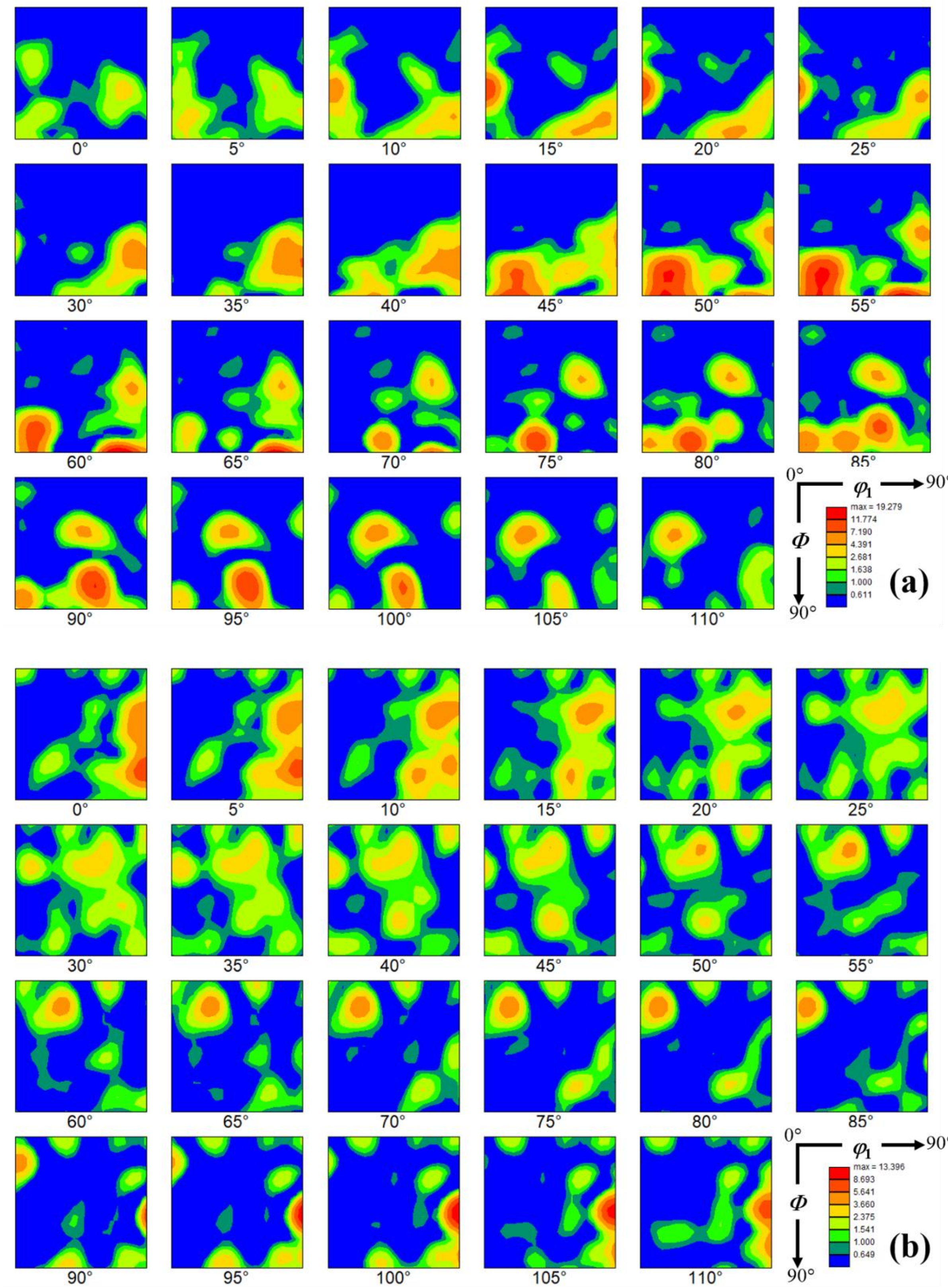
0°
5°
10°
15°
20°
25°
30°
35°
40°
45°
50°
55°
60°
65°
70°
75°
80°
85°
90°
95°
100°
105°
110°
0°
φ_1
90°
Φ
90°
max = 19.279
11.774
7.190
4.391
2.681
1.638
1.000
0.611
(a)
0°
5°
10°
15°
20°
25°
30°
35°
40°
45°
50°
55°
60°
65°
70°
75°
80°
85°
90°
95°
100°
105°
110°
0°
φ_1
90°
Φ
90°
max = 13.396
8.693
5.641
3.660
2.375
1.541
1.000
0.649
(b)

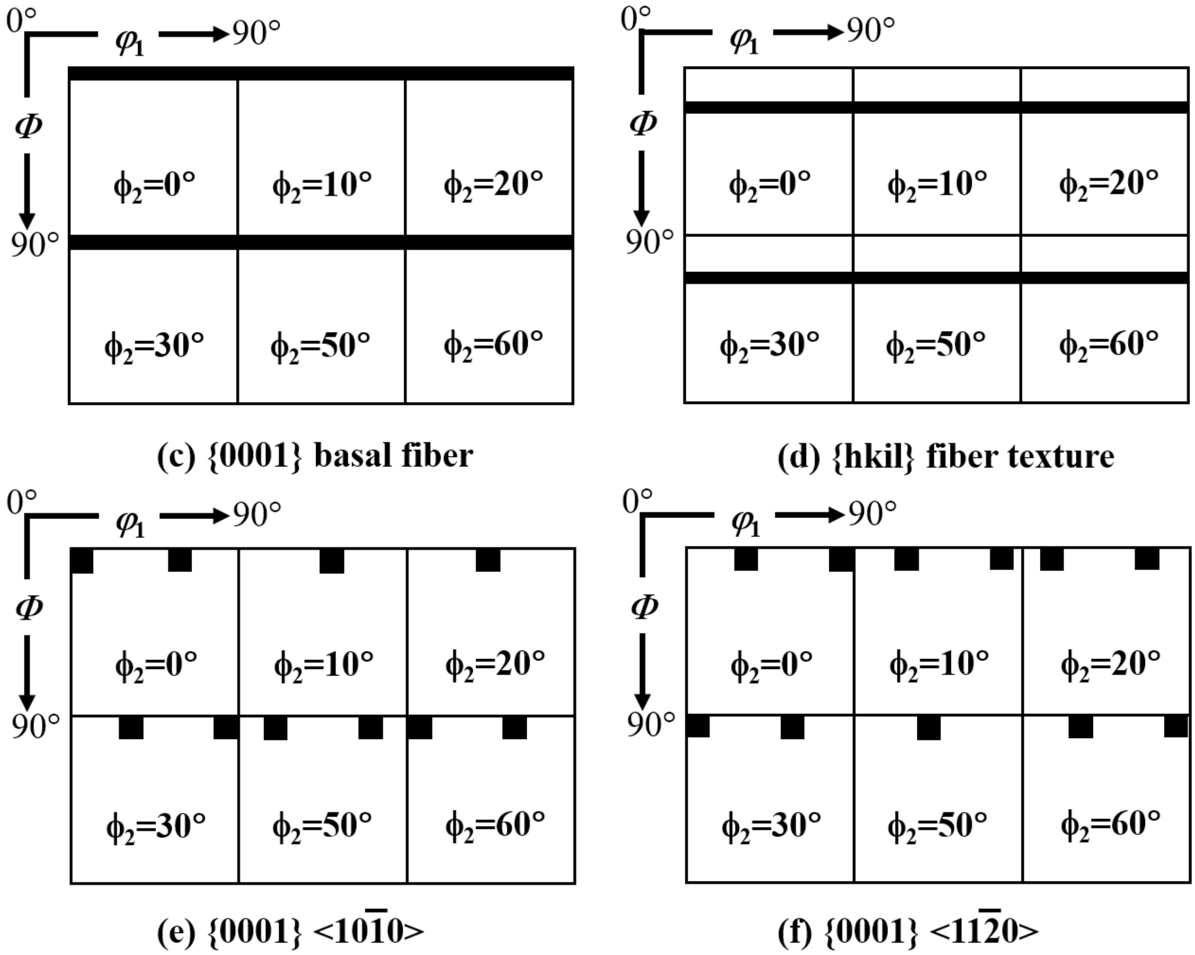

Figure S7. Orientation distribution function (ODF) patterns for the a) HD-0Te sample and b) LPHD-16Te sample. The ideal ODF sections for some low index orientation of the hexagonal close packed (HCP) crystalline structure when forming c) {0001} basal fiber, d) {hkil} fiber texture, e) {0001}<10$\bar{1}$0> texture and f) {0001}<11$\bar{2}$0> texture.[2]

Orientation distribution function (ODF) is an effective method to precisely describe the texture components in a three-dimensional Euler orientation space. An entire ODF picture is composed of many ODF sections with different ϕ_2 value, as shown in Figure S7a and S7b. The ideal ODF sections for HCP structure (Figure S7c, S7d, S7e and S7f) exhibit that although ODF patterns differs by the texture type and texture orientation, highlighted regions will always locate at the top

of ODF sections ($\Phi = 0$) if the crystal has a (000*l*) preferred orientation.[2] Based on this, the (000*l*) texture is quite weak in the HD-0Te sample (see Figure S7a) and is strongly enhanced in the LPHD-16Te alloys (see Figure S7b**)**. In addition, the highlighted regions with $\Phi = 0$ in Figure S7b fit relatively well with Figure S7e, indicating the formation of $(000l)\langle 10\bar{1}0\rangle$ rolling texture in the LPHD-16Te sample.

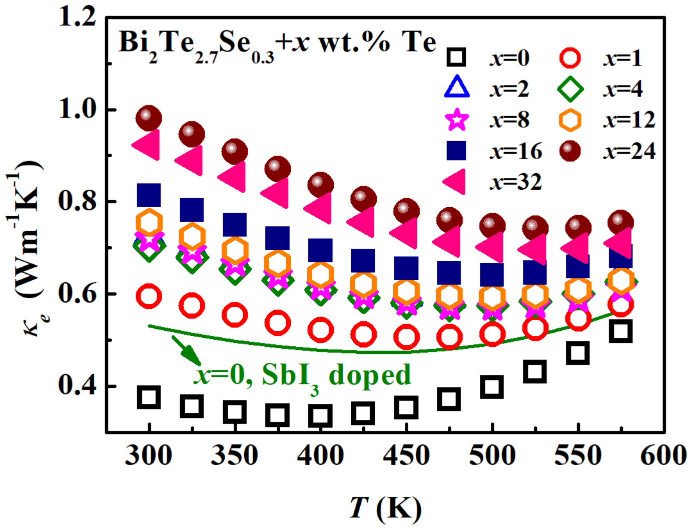

Figure S8. Temperature dependences of in-plane electronic thermal conductivity for the HD-0Te sample and LPHD-*x*Te samples. The green curve is the HD $Bi_2Te_{2.7}Se_{0.3}$ sample with SbI_3 doping.

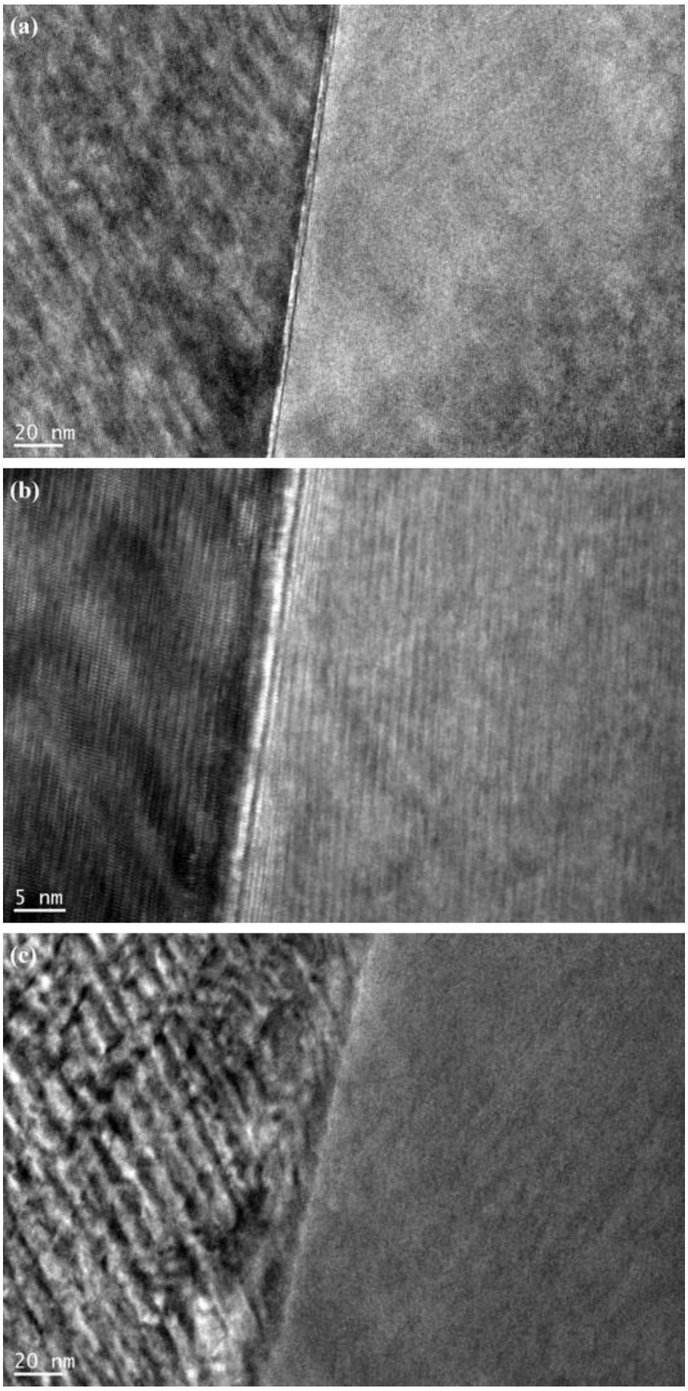

Figure S9. Grain boundary images of LPHD-16Te sample by tilting the TEM zone axis.

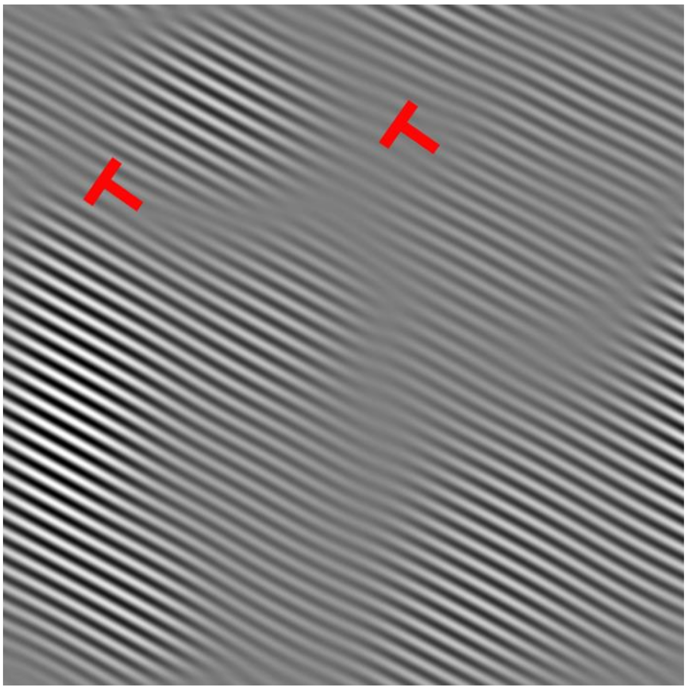

Figure S10. Inverse FFT image of the blue box in Figure 11(c) obtained from (015) reflection.

Table S1. EDS analysis of spots in Figure 2a and 2b (at.%).

element	#1	#2	#3	#4	#5	#6	#7	#8	#9
**Bi**	2.07	2.03	1.58	1.41	1.67	1.52	1.84	0	0
**Te**	2.55	2.67	3.30	3.50	3.24	3.39	3.06	1	1
**Se**	0.38	0.30	0.12	0.08	0.09	0.09	0.10	0	0

Table S2. EPMA analysis of spots in Figure S1 (at.%).

**region**		**spots**	**actual composition**	**normally average composition**
brighter region		#1	$Bi_{2.04}Te_{2.76}Se_{0.20}$	$Bi_2Te_{2.66}Se_{0.26}$
		#2	$Bi_{2.02}Te_{2.75}Se_{0.23}$	
		#3	$Bi_{2.02}Te_{2.65}Se_{0.33}$	
		#4	$Bi_{2.04}Te_{2.62}Se_{0.35}$	
		#5	$Bi_{2.03}Te_{2.73}Se_{0.24}$	
		#6	$Bi_{2.04}Te_{2.73}Se_{0.22}$	
		#7	$Bi_{2.03}Te_{2.64}Se_{0.33}$	
		#8	$Bi_{2.02}Te_{2.74}Se_{0.24}$	
darker region	Te-rich phase	#9	Te	Te
		#10	Te	
		#11	Te	
		#12	Te	
	Te-poor phase	#13	$Bi_{1.32}Te_{3.60}Se_{0.07}$	$Bi_2Te_{4.30}Se_{0.10}$
		#14	$Bi_{1.34}Te_{3.60}Se_{0.06}$	
		#15	$Bi_{1.71}Te_{3.21}Se_{0.08}$	
		#16	$Bi_{1.85}Te_{3.05}Se_{0.10}$	
		#17	$Bi_{1.59}Te_{3.33}Se_{0.08}$	

References

[S1] a) L. P. Hu, H. J. Wu, T. J. Zhu, C. G. Fu, J. He, P. J. Ying, X. B. Zhao, *Adv. Energy Mater.* **2015**, 5, 1500411; b) Y. H. Wu, R. S. Zhai, T. J. Zhu, X. B. Zhao, *Mater. Today Phys.* **2017**, 2, 62; c) H. Cho, J. H. Kim, S. Y. Back, K. Ahn, J. S. Rhyee, S. D. Park, *J. Alloys Compd.* **2018**, 731, 531.

[S2] Y. N. Wang, J. C. Huang, *Materials Chemistry and Physics* **2003**, 81, 11.
